# Enzymatic/Mitochondrial Import Bottleneck in Sepsis with Possible Extensions to DIC: IV Glutathione and Hinokitiol as Potential Therapeutic Candidates

**DOI:** 10.3390/ijms27198642

**Published:** 2026-09-27

**Authors:** Felix Badiu, Mark Slevin

**Affiliations:** 1 Faculty of Medicine, George Emil Palade University of Medicine, Pharmacy, Science and Technology of Târgu Mureș, 540139 Târgu Mureș, Romania; badiu.felix.24@stud.umfst.ro; 2 Centre for Advanced Medical and Pharmaceutical Research, George Emil Palade University of Medicine, Pharmacy, Science and Technology of Târgu Mureș, 540139 Târgu Mureș, Romania

**Keywords:** Sepsis, DIC, Glutathione, *GCLC*, *NLRP3*, *GSDMD*, *SLC25A39*, Mitophagy, *BNIP3L*/*FUNDC1*, xCell

## Abstract

Sepsis-induced disseminated intravascular coagulation (DIC) carries approximately 44% mortality and has no targeted mechanistic therapy. Stable isotope tracer data show whole-blood glutathione (GSH) synthesis falling by approximately 60% in sepsis despite paradoxically elevated cysteine flux, consistent with enzymatic failure at the glutamate-cysteine ligase (GCL) step rather than substrate shortage. We propose that GCL failure may unify endothelial coagulopathy and innate immune dysfunction in DIC, linked to the *NLRP3*/*GSDMD* pyroptotic coagulation pathway through both cytosolic synthesis and mitochondrial import failure. Cross-dataset bioinformatic analysis was conducted across four independent GEO datasets. Three whole-blood cohorts (n = 200; 144 sepsis, 56 controls; two platforms) were selected for this study (two adult cohorts and one pediatric cohort). All cohorts were strictly filtered to day 1 baseline samples to prevent pseudoreplication. Differential expression was evaluated via Mann–Whitney U with Benjamini–Hochberg correction, with xCell deconvolution resolving bulk signals into cell-type-specific components via Spearman’s correlation. Mitochondrial GSH transporter and receptor-mediated mitophagy genes were evaluated using the same pipeline as an independent evidential layer. A fourth dataset of isolated monocytes (GSE46955; n = 14 individuals, 22 samples) was analyzed to assess *AFG3L2* and *SLC25A39* co-transcription. Across cohorts, *NLRP3* transcript abundance positively correlated with monocyte and neutrophil enrichment scores (e.g., rho = +0.633 and +0.537). Conversely, transcripts for *GCLC* (rho = −0.530) and *SLC25A39* (rho = −0.400), the principal mitochondrial GSH importer, inversely correlated with these same myeloid population scores. Furthermore, the receptor-mediated mitophagy transcripts *FUNDC1* and *BNIP3L* were significantly downregulated and inversely correlated with expanding myeloid fractions. These bulk transcriptomic patterns reveal a strong inverse relationship between the expression of inflammasome components and GSH synthesis, import, and clearance machinery, though further studies are required to confirm functional protein activation or enzymatic failure. Longitudinal analysis of isolated monocytes, utilizing a linear mixed-effects model to account for repeated within-patient sampling, demonstrated significant aggregate co-transcription between *AFG3L2* and *SLC25A39* (*p* = 0.012), indicating tight co-regulation of the importer and its protease. These findings, derived from sepsis-versus-control cohorts without DIC adjudication, suggest that the transcriptional suppression of GSH synthesis and mitochondrial import machinery correlates strongly with *NLRP3* and *GSDMD* transcript upregulation in estimated myeloid populations. Extending this axis to a mechanistic role in DIC is a hypothesis supported by the independent literature, not a direct finding of the present dataset. Because this bottleneck appears enzymatic rather than substrate-dependent, N-acetylcysteine alone may be insufficient to restore homeostasis. We therefore hypothesize that combining intravenous glutathione with an Fe–S cluster-promoting agent, such as hinokitiol, could mechanistically address these findings. However, this remains strictly a preclinical hypothesis that requires extensive investigation to resolve existing safety concerns, particularly the potential for ionophore toxicity in humans.

## 1. Introduction

Sepsis is defined by life-threatening organ dysfunction caused by a dysregulated host response to infection, wherein the body’s own defence mechanisms become the primary source of tissue damage rather than the pathogen itself [1]. Among its complications, disseminated intravascular coagulation represents one of the most feared, developing in 30–50% of septic shock patients and, once established in its overt form, carrying a mortality of approximately 44% [2,3]. Despite decades of investigative effort, no intervention currently targets the mechanism driving this coagulopathy. Supportive measures, including fresh frozen plasma, platelet transfusion, and anticoagulation, address the consequences of DIC rather than its cause.

Tracing the pathological sequence suggests that endothelial cell death may be the primary instigator of the cascade. Because the microvasculature fails first, it directly drives the subsequent coagulation abnormalities, which induce thrombin generation, protein C pathway collapse, and fibrinolytic suppression, all manifesting as secondary consequences of this initial endothelial collapse [4,5]. The principal defence against the oxidative stress driving endothelial cell death is glutathione, the cell’s predominant intracellular antioxidant, which is consistently and profoundly depleted in sepsis [6]. However, the mechanistic basis of this depletion, and its precise connection to coagulation activation, has implications that have not yet been fully described or characterised.

We propose that systemic glutathione depletion occurs independently of biosynthetic precursor availability. Instead, the septic environment drives four converging pathological mechanisms that systematically inactivate glutamate-cysteine ligase (GCL), the rate-limiting enzyme for GSH synthesis. GCL is a heterodimer of a catalytic subunit, *GCLC*, and a modifier subunit, *GCLM*, the latter of which governs holoenzyme efficiency and emerges consistently as the rate-limiting determinant of biosynthetic output [7]. Because the metabolic bottleneck is inherently enzymatic, we hypothesize that precursor supplementation with N-acetylcysteine may prove insufficient. Instead, bypassing the inactivated ligase through direct intravenous glutathione repletion may represent a necessary prerequisite for restoring cellular homeostasis, addressing the metabolic deficit directly. This hypothesis is intended to be confirmed by this paper’s bioinformatic analysis; however, in vitro or in vivo validation is required.

Furthermore, we hypothesize that the septic environment compromises mitochondrial glutathione importation by altering the expression profile of key mitochondrial transporters. Initially, as demonstrated across multiple transcriptomic datasets, transcripts for the importer *SLC25A39* and its regulatory protease *AFG3L2* undergo acute co-downregulation, suggesting a transcriptionally driven reduction in baseline import capacity. In an independent isolated-monocyte cohort, *AFG3L2* and *SLC25A39* similarly showed significant aggregate co-transcription, indicating that the importer and its protease are tightly co-regulated at the transcript level. If newly synthesized *SLC25A39* remains vulnerable to *AFG3L2*-mediated degradation under conditions of sepsis-induced Fe–S cluster depletion, direct glutathione repletion might be therapeutically limited unless combined with agents that promote Fe–S cluster stability to armor the nascent transporter. This hypothesis is supported by transcriptomic analysis of 144 unique baseline sepsis patients comprising 99 pediatric patients (GSE13904), 35 adult septic shock patients (GSE54514), and 10 adult ICU sepsis patients (GSE28750). The myeloid cells with the highest *NLRP3* transcript levels were precisely the cells in which *GCLC* expression was most reduced, and *SLC25A39*, the transporter responsible for importing cytoplasmic glutathione into the mitochondrial matrix, was simultaneously downregulated in the same cellular compartment. This three-level mechanistic convergence, comprising the coordinated transcriptional suppression of glutathione synthesis and mitochondrial import machinery alongside elevated inflammasome transcript abundance within estimated myeloid populations, constitutes the central original hypothesis of this paper and has not previously been described.

## 2. Results

### 2.1. Bulk Differential Expression

Our analysis identified *NLRP3* as showing significant directional upregulation in the pediatric cohort (log2FC = +0.295; *p*.adj. < 0.0001), alongside modest bulk alterations in *GCLC* and *GCLM* across the datasets. While *GCLC* bulk changes were variable, *GCLM* exhibited striking bulk transcriptional upregulation in two of the datasets (log2FC = +1.522 to +1.964; *p*.adj. < 0.001). However, because whole-blood expression profiles are vulnerable to leukocyte redistribution during sepsis, this apparent bulk upregulation of *GCLM*, as well as the specific cellular tracking of *GCLC* and *NLRP3*, must be resolved through cell-type deconvolution, as detailed in Section 2.2. Full results for all targets are presented in Table 1 GSE28750 results (n = 10) are treated as directional confirmation only.

### 2.2. Cross-Dataset Consistency and Deconvolution Resolution

Cell-type-specific deconvolution of bulk whole-blood transcriptomics yielded five critical mechanistic insights that resolve the inherent noise of systemic sepsis data.

#### 2.2.1. *NLRP3* Upregulation Is Concentrated in Myeloid Cells

We identified robust positive correlations between *NLRP3* expression and estimated monocyte abundance in the pediatric cohort (rho = +0.633 in GSE13904; adjusted *p* < 0.0001), suggesting that elevated inflammasome transcription is strongly associated with these myeloid populations. Notably, the lack of bulk significance in GSE54514 (adjusted *p* = 0.408), despite this massive cell-specific correlation, perfectly illustrates the absolute necessity of deconvolution. Bulk whole-blood significance is subject to the highly variable monocyte fraction per patient, which independently masks the strong correlation between *NLRP3* transcription and myeloid enrichment.

#### 2.2.2. *GCLM* Bulk “Upregulation” as a Compositional Artifact

The apparent bulk upregulation of *GCLM* is consistent with a compositional artifact arising from the well-documented myeloid-to-lymphocyte ratio shift in acute sepsis, rather than reflecting genuine compensatory transcription. Monocyte, neutrophil, and macrophage fractions are significantly elevated in sepsis (GSE13904 monocytes, Wilcoxon median delta = +0.413, *p*.adj. = 4.20 × 10^−6^; neutrophils, delta = +0.460, *p*.adj. = 4.20 × 10^−6^; M1 macrophages, delta = +0.079, *p*.adj. = 7.68 × 10^−6^), while CD8+ T-cell, CD4+ T-cell, and B-cell fractions are significantly reduced (GSE13904 CD8+ T-cells, delta = −0.377, *p*.adj. = 4.20 × 10^−6^; B-cells, delta = −0.601, *p*.adj. = 5.05 × 10^−5^), consistent with well-described sepsis-associated myeloid expansion and lymphopenia, and matched by *GSDMD*’s positive correlation with the same expanding myeloid populations (Table 2).

#### 2.2.3. *GCLC* Transcript Abundance Inversely Correlates with Estimated Myeloid Fractions

The primary catalytic subunit *GCLC* exhibits an inverse relationship with estimated myeloid abundance, correlating negatively with estimated monocyte scores in the adult cohort (GSE54514 Spearman’s rho = −0.530; *p*.adj. = 0.00048). An inverse association is similarly observed in the neutrophil compartment of the independent pediatric cohort (GSE13904 Spearman’s rho = −0.340; *p*.adj. = 0.0016). These cross-platform correlations indicate that lower bulk *GCLC* transcript abundance statistically tracks with the expansion of circulating myeloid fractions. Cross-platform, cross-cohort convergence on the same two myeloid populations was identified across the two different arrays. Crucially, the opposed statistical correlations of these pathways across independent platforms, with *NLRP3* expression tracking strongly with estimated monocyte abundance in GSE13904 (rho = +0.633) while *GCLC* correlates negatively with monocyte estimates in GSE54514 (rho = −0.530), suggest that elevated inflammasome signaling and suppressed glutathione transcription are both highly associated with the presence of these circulating myeloid subsets. Notably, this relationship persists even in cohorts where the overall estimated monocyte fraction does not significantly differ from controls.

#### 2.2.4. *GSDMD* Transcript Levels Are Elevated in Multiple Myeloid and Lymphoid Populations

The pyroptotic executor *GSDMD* presents a highly conflicted bulk signal: modestly significant in GSE13904 (log2FC = +0.087; *p*.adj. = 0.048) and null in GSE54514. Cell-type deconvolution resolves part of this conflict: in GSE13904, *GSDMD* shows significant positive correlations with estimated M1 macrophage (rho = +0.379, *p*.adj. < 0.001) and neutrophil (rho = +0.253, *p*.adj. < 0.05) populations. This indicates that *GSDMD* transcript elevation is most strongly associated with these specific estimated myeloid compartments during pediatric sepsis, though it lacks significant replication in the adult cohorts.

#### 2.2.5. Receptor-Mediated Mitophagy Transcripts Are Suppressed in the Sepsis Landscape

To evaluate the status of mitochondrial clearance mechanisms, we checked the baseline expression of both classical and receptor-mediated mitophagy genes. The classical *PRKN* (Parkin) transcript exhibited highly volatile expression situated near the background noise level across the bulk whole-blood arrays, precluding reliable analysis. Conversely, the Parkin-independent mitophagy receptors *BNIP3L* (NIX) and *FUNDC1* demonstrated robust, consistent baseline detection. In bulk differential expression analysis, *FUNDC1* exhibited highly consistent transcriptional downregulation across all three independent whole-blood sepsis cohorts (log2FC = −0.154 to −1.150; all *p*.adj. < 0.05). Upon cell-type deconvolution, lower *FUNDC1* transcript abundance strongly correlated with the expansion of estimated M2 macrophage populations (GSE13904 rho = −0.501, GSE28750 rho = −0.840). Concurrently, while *BNIP3L* presented a mixed bulk signal, its transcript abundance inversely correlated with estimated circulating monocytes (GSE54514 rho = −0.451, GSE13904 rho = −0.271) and neutrophils (GSE13904 rho = −0.310) (Table 3). This widespread downregulation of *FUNDC1* in bulk datasets, paired with the inverse relationship between both receptor transcripts and expanding myeloid fractions, indicates that the transcriptional components essential for receptor-mediated mitochondrial clearance are notably deficient in the septic state.

### 2.3. Mechanistic Convergence in Myeloid Cells

The synthesis and transporter analyses establish multiple independent levels of evidence consistently pointing to the same cellular compartment. *GCLC* was inversely correlated with monocytes and neutrophils (rho = −0.530 to −0.340, cross-platform, confirmed in two larger datasets) (Figure 1). *SLC25A39*, recently identified by Wang et al. [8] as the primary mammalian mitochondrial glutathione (mGSH) importer, was consistently downregulated across all three whole-blood datasets (significant in GSE13904; adjusted *p* = 0.0071). Upon deconvolution, *SLC25A39* transcript abundance exhibited a robust inverse correlation with estimated myeloid enrichment scores (rho = −0.400 in monocytes and −0.230 in M1 macrophages, both in GSE13904). Mechanistically, Shi et al. demonstrated that the mitochondrial m-AAA protease *AFG3L2* constitutively degrades *SLC25A39* via its matrix loop 1 domain. This degradation is regulated by an iron–sulfur (Fe–S) cluster-sensing mechanism localized to four matrix-facing cysteine residues on *SLC25A39*; when Fe–S clusters are abundant, degradation is inhibited, directly coupling mitochondrial glutathione import capacity to iron–sulfur homeostasis [9].

### 2.4. Within-Patient AFG3L2–SLC25A39 Co-Transcription in Isolated Monocytes

Because GSE46955 contains repeated acute and recovery-phase samples from the same eight patients, a correlation across all 22 samples would violate the independence assumption underlying a standard significance test. We therefore modeled *SLC25A39* transcript abundance as a function of *AFG3L2* transcript abundance using a linear mixed-effects model (*SLC25A39* ~ *AFG3L2* + (1|patient), REML) with a random intercept per patient. This model confirmed a significant positive within-patient relationship between *AFG3L2* and *SLC25A39* (β = 0.404, SE = 0.140, t(13.5) = 2.89, *p* = 0.0122), consistent in direction with a naive Spearman correlation computed across all 22 samples without correcting for repeated measures (rho = 0.540, *p* = 0.0105) but now properly attributable to within-patient co-regulation rather than to between-patient differences alone. While we attempted to further stratify this relationship into distinct acute and recovery sub-models, the small number of patients (n = 8) did not support a reliable additional stratification. Ultimately, the primary observable defect remains the profound baseline transcriptional downregulation of *SLC25A39* established in the acute whole-blood cohorts. Kawase et al. examined *SLC25A39/40* expression through in vivo models using murine hepatocytes and whole livers from bile duct ligation and intraperitoneal LPS-treated mice, wherein LPS exposure induced a highly significant and targeted upregulation of *SLC25A40* mRNA specifically within the kidneys. Using human clear cell renal carcinoma (KMRC-1) cells treated with LPS-RS to block Toll-like receptor 4 (TLR4) and *FPS-ZM1* to inhibit the receptor for advanced glycation end-products (RAGE), they successfully and completely suppressed the LPS-induced expression of *SLC25A40*. Ultimately, this mechanistic validation confirmed that the upregulation of *SLC25A40* was driven through the *TLR4/RAGE* signalling axis rather than reflecting a homeostatic redox response [10].

Using siRNA-mediated *SLC25A40* knockdown in murine bone marrow-derived macrophages, Zheng et al. demonstrated that this compensatory upregulation was functionally insufficient. The study showed that even in the presence of LPS-induced *SLC25A40* upregulation, reduced *SLC25A40* expression destabilised iron–sulfur cluster-rich electron transport chain proteins, elevated both mitochondrial and cellular ROS, and, paradoxically, induced compensatory transcriptional upregulation of *GCLC* and *GCLM*, confirming that *SLC25A40* upregulation was insufficient to prevent ETC destabilisation and mitochondrial ROS accumulation. Furthermore, *SLC25A40* deficiency diminished IL-1β production following *NLRP3* activation by nigericin or ATP, underlining a direct mechanistic link between mitochondrial GSH import failure and impaired inflammasome-driven cytokine output [11].

In sepsis, the same LPS-TLR4 signalling event that drives the pro-inflammatory cascade and *NLRP3* priming simultaneously triggers a compensatory *SLC25A40* upregulation. The transcriptomic profile suggests a compensatory response to mGSH depletion. However, this compensation would occur against a background of simultaneous GCL suppression from other mechanisms characteristic of sepsis, *SLC25A39* transcriptional downregulation, and *AFG3L2*-mediated post-translational destabilisation of the primary GSH importer. Therefore, the *SLC25A40* response may be structurally incapable of restoring mGSH homeostasis. *NLRP3* transcript abundance was strongly positively correlated with estimated monocyte and neutrophil populations (rho = +0.537 to +0.633), the same cells with *GCLC* and *SLC25A39* defects. The probability that the transcriptional suppression of synthesis and import machinery, alongside inflammasome transcription upregulation, all correlate strongly with the exact same estimated myeloid compartments across independent cohorts by chance is low. Causal direction requires experimental testing.

## 3. Discussion

### 3.1. GCL Modulation and GSH: Is This the Enzymatic Bottleneck?

The enzymatic pathway of glutathione synthesis, which tends to be overlooked, could play a crucial role in the onset of DIC and the progression of sepsis. Furthermore, it may indicate why some therapeutic interventions might fail, despite the presence of abundant substrate.

Glutathione is synthesised in two sequential ATP-dependent steps. The first step is represented by the formation of γ-glutamylcysteine from L-glutamate and L-cysteine, catalyzed by the enzyme glutamate-cysteine ligase GCL, a heterodimer comprising a catalytic subunit (*GCLC*) and a modifier subunit (*GCLM*). Studies conducted on rat liver and HepG2 hepatocytes cultured in low-sulphur amino acid-containing medium demonstrated that the association of *GCLC* with *GCLM* increased both GCL affinity and overall activity. Whilst *GCLC* represents the catalytic core, *GCLM* expression consistently emerged as the rate-limiting bottleneck for GSH synthesis; *GCLM*-deficient mice exhibited tissue GSH levels of only 9–16% of normal, despite compensatory upregulation of *GCLC* [7]. The second step is mediated by glutathione synthetase, which catalyzes the synthesis of GSH from γ-glutamylcysteine and glycine; overexpressing glutathione synthetase yields no appreciable change [12].

Nrf2 (nuclear factor erythroid 2-related factor 2), a direct mediator of GCL and *GCLM* expression, is an important indicator that could determine the severity of sepsis and potentially mortality. Supporting this, Nrf2 gene knockout studies in a mouse model showed that disruption of Nrf2 signalling markedly increased the mortality rate, whilst concomitantly increasing LPS and TNF-alpha production, culminating in significantly greater lung inflammation as early as 30 min after i.p. injection with a lethal dose of LPS. High levels of Nrf2 in the lungs confirmed a proportional increase of glutathione reductase-1 over time [13]. Furthermore, activation of the Nrf2 pathway through genetically deleting its repressor protein, *Keap1*, in myeloid cells showed decreased pro-inflammatory cytokine levels, reduced bacteraemia, and improved survival in the same model system. In contrast, Nrf2-deficient macrophages were hypersensitive to LPS because of the accumulation of ROS [14].

Under basal conditions, Nrf2 is constitutively sequestered in the cytoplasm through its binding to *Keap1*, a substrate recognition adaptor for a CUL3-based E3 ubiquitin ligase complex that targets Nrf2 for proteasomal degradation. Itoh et al. identified this repressive interaction through yeast two-hybrid screening and co-immunoprecipitation assays in transfected COS1 cells, demonstrating that *Keap1* bound specifically to the amino-terminal Neh2 regulatory domain of Nrf2 and suppressed antioxidant response element-mediated transcription of phase II detoxifying enzyme genes, including those encoding GCL subunits. Rising levels of oxidative stress may relieve this repression through oxidative modification of reactive cysteine sensor residues within the *Keap1* BTB and intervening region domains, which impairs the ability of the *Keap1*-CUL3 complex to ubiquitinate Nrf2, allowing it to accumulate and translocate to the nucleus where it would upregulate transcription of both *GCLC* and *GCLM* through antioxidant response element binding, restoring the biosynthetic machinery required for GSH production [15]. This compensatory dissociation may be sufficient to maintain redox homeostasis in early sepsis. However, the sustained inflammatory environment of severe sepsis may progressively overwhelm and suppress Nrf2 transcriptional activity through mechanisms including NF-κB-mediated competitive inhibition and post-translational modification of Nrf2 itself.

Multiple converging mechanisms are thought to suppress GCL activity within the septic environment. Notably, studies in rat hepatocytes, H4IIE rat hepatoma cells, and Huh-7 human hepatoma cells demonstrated that endotoxaemia markedly reduced both *GCLC* and *GCLM* mRNA and protein levels by 50–60%, with near-maximal suppression occurring as early as six hours following LPS administration. The remaining monomeric *GCLC* retained intrinsic enzymatic activity but operated at a fraction of its normal rate [16]. Skeletal muscle biopsies on 28 septic patients and nine patients undergoing elective hip surgery showed that mitochondrial complex I dysfunction reduced ATP supply to the GSH synthesis reaction. Complex I activity inversely correlated with SOFA score and 28-day mortality in septic shock [17], quantitatively linking the collapse of cellular energy to clinical outcome.

This suggests that even where enzymatic machinery remains intact, the bioenergetic environment of severe sepsis may hinder GSH biosynthesis at both catalytic steps. In vitro models using Jurkat T lymphocytes and HeLa cells showed that caspase-3 protease was activated as endothelial cells began to die, directly cleaving *GCLC* at the Asp499 site, producing a non-functional 60 kDa fragment and simultaneously triggering extrusion of the cell’s remaining GSH stores [18]. This last mechanism is particularly important because it propagates failure: as early dying cells extrude GSH and activate caspase-3, adjacent cells may be drawn into the same cascade before their own mitochondria have been damaged.

Using stable isotope tracer methodology to study whole-blood ICU-admitted pediatric patients, specifically intravenous infusion of labelled cysteine with gas chromatography–mass spectrometry, Lyons et al. demonstrated that whole-blood GSH synthesis rates fell by approximately 60% in septic patients while plasma cysteine flux was simultaneously and paradoxically elevated above controls (68.2 ± 17.5 vs. 48.7 ± 8.8 micromol/kg/h, *p* < 0.01) [19]. Although more substrates were available, significantly fewer products were made.

These findings might partially redefine the mechanistic basis of GSH depletion in sepsis; the limiting factor appears not to be the availability of precursor substrate, but rather the active enzymatic suppression of the biosynthetic machinery itself within the septic environment.

### 3.2. Converging Pathways to DIC

The mitochondrial consequences of GCL failure represent a pathway through which enzymatic GSH depletion could initiate the coagulopathy of DIC. Selective mitochondrial GSH (mGSH) depletion has been shown to disproportionately impair endothelial inflammatory regulation relative to cytoplasmic GSH loss. In chronic ethanol-fed hepatocytes, defective mitochondrial GSH transport produced a 65% fall in mGSH against only a 24% fall in cytoplasmic GSH [20]. In HUVECs, mGSH reduction below 15% of control markedly enhanced TNF-α-induced adhesion molecule expression and leukocyte adhesion in a way cytoplasmic depletion alone did not [21]. These results establish mGSH as a functionally specific checkpoint for endothelial inflammatory responses. This is consistent with the anatomical pattern of DIC itself, which preferentially targets the high-mitochondrial-density microvascular endothelium of lung, kidney, liver, and gut.

#### 3.2.1. Receptor-Mediated Mitophagic Failure (*BNIP3L*/*FUNDC1*) and Procoagulant Endothelial Apoptosis

As established in Section 2.2.5, the PRKN transcript exhibited volatile expression near background noise levels across the whole-blood cohorts. Conversely, the receptor-mediated mitophagy transcripts *BNIP3L* and *FUNDC1* demonstrated robust baseline detection but were found to be significantly downregulated across the septic cohorts. The mechanistic discussion below extrapolates from the transcriptional suppression observed in our cohorts to the functional mitophagic failure established by independent experimental models. Unlike *PINK1/Parkin*, *BNIP3L/NIX* and *FUNDC1* recruit the autophagosome directly via their own LC3-interacting motifs, gated by phosphorylation state, and are activated under hypoxia and energy deficiency [22,23] conditions already established for the septic endothelium in Section 3.1. Critically, *BNIP3L/NIX* also carries an atypical BH3-like domain that independently antagonises *Bcl-2/Bcl-xL*, licensing Bax/Bak-mediated cytochrome c release [24]—the same molecule that fails to clear a damaged mitochondrion also primes it for apoptosis once clearance fails. Direct support in septic endothelium comes from Gao et al., who showed that *FUNDC1*-mediated mitophagy in cardiac microvascular endothelial cells becomes insufficient in later-stage LPS injury, allowing damaged mitochondria to accumulate, drive apoptosis, and impair measured endothelial barrier function [25]. This is the closest existing experimental analogue to the endothelial arm proposed here, though it derives from cardiac rather than pulmonary, renal, or hepatic microvasculature, and reflects sepsis-induced organ dysfunction. Therefore, extrapolation to the vascular beds implicated in DIC remains an assumption, not a demonstrated finding.

In sepsis, this protective pathway is suppressed through two simultaneous mechanisms, both connecting directly to GCL failure. Firstly, constitutively active mTORC1, driven by the sustained inflammatory environment, suppresses the ULK1 complex that initiates autophagy [26].

Studies conducted in HEK293 cells and primary murine hepatocytes demonstrated that mTOR inhibition restored mitophagic capacity following mitochondrial uncoupling, confirming that inflammatory mTORC1 activation was sufficient to disable the mitophagic response [27]. When *BNIP3L*/*FUNDC1*-mediated clearance fails under these conditions, accumulated damaged mitochondria are expected to commit the cell to apoptosis through BH3-driven Bax/Bak conformational activation, outer-membrane permeabilisation, cytochrome c release, and apoptosome-dependent caspase-9 activation [28]. While our whole-blood transcriptomic data cannot directly confirm endothelial cell fate, independent studies have indicated that suppression of mitophagy initiation and execution leads to the accumulation of damaged mitochondria. Consequently, we infer that this sequence would trigger cytochrome c release, committing the microvascular endothelium to apoptosis.

Therefore, this failure is likely to be the principal trigger for subendothelial surface exposure and procoagulant microparticle shedding via phosphatidylserine externalisation. GCL failure, through both cytoplasmic GSH depletion and mitochondrial cytochrome c exposure, would thus mechanistically link to the coagulation cascade rather than arising as a parallel consequence of sepsis severity.

Supporting this, studies examining procoagulant microparticle generation in plasma from patients with fulminant meningococcal sepsis and DIC, flow cytometric and immunoprecipitation analyses identified elevated numbers of circulating CD14-positive, tissue factor-positive microparticles, with 85% of all TF-positive microparticles bearing the CD14 monocyte surface marker, establishing monocyte-derived microparticles as the primary source of procoagulant TF activity in the septic circulation.

In a case study of a patient with severe DIC, the number of endothelial cell-derived microparticles was disproportionately elevated compared with the remainder of the cohort, and plasma procoagulant activity measured by in vitro thrombin generation was markedly enhanced, consistent with microparticle-driven coagulation initiation independent of intact endothelial apoptosis [29].

Endothelial tissue factor expression was observed in LPS-treated mice and rabbits at the protein level, and TF co-localisation with the endothelial marker von Willebrand factor was demonstrated in the splenic microvasculature of septic baboons; however, a substantial proportion of apparent endothelial TF signal was attributable to leukocyte-derived microparticles docking onto activated endothelial surfaces rather than de novo endothelial synthesis, and direct in vivo evidence of endothelial TF expression in humans remains limited [30].

Consequently, the simultaneous exposure of subendothelial collagen and tissue factor forces the activation of the extrinsic pathway. This widespread conversion of fibrinogen to fibrin across the capillary beds of the lung, kidney, liver, and gut aligns precisely with the anatomical distribution of DIC end-organ failure [31].

The downstream coagulation consequences of endothelial apoptosis were studied across three complementary bodies of evidence. In human aortic endothelial cells (HAECs) subjected to LPS stimulation in vitro, Ye et al. demonstrated that LPS signalling directly upregulated *PAI-1* expression and suppressed thrombomodulin surface display, establishing the molecular basis for the simultaneous loss of fibrinolytic and anticoagulant capacity that defines the endothelial phenotype of septic DIC [32]. Thrombomodulin, an integral membrane glycoprotein expressed on the surface of endothelial cells, is essential for thrombin-mediated activation of protein C, a physiological anticoagulant. As endothelial cells die and shed their surface, thrombomodulin is stripped from the vascular surface, rendering the protein C pathway non-functional regardless of circulating protein C levels [4]. Concurrently, *PAI-1*, released from damaged endothelium, inhibited both tissue-type and urokinase-type plasminogen activators, suppressing fibrinolysis and allowing fibrin microthrombi to accumulate unchecked across the microvasculature. In a clinical observational cohort of sepsis-associated DIC patients, it was observed that soluble thrombomodulin and *PAI-1* levels correlated directly with DIC severity and organ failure, with *PAI-1* peaking at sepsis onset and reflecting the intensity of both inflammation and coagulopathy in the early phase, whilst soluble thrombomodulin release into plasma served as a quantitative marker indicative of endothelial surface destruction [5].

The resulting laboratory phenotype, comprising elevated D-dimer from fibrin degradation products, hypofibrinogenaemia, prolonged PT and APTT, and thrombocytopenia, represented the arithmetic consequence of this simultaneous procoagulant acceleration and anticoagulant failure, with bleeding from factor consumption and microvascular thrombosis from fibrin deposition occurring in parallel [4,5,32].

#### 3.2.2. Mitochondrial Collapse Drives Coagulation Activation via the *NLRP3*/*GSDMD* Pyroptotic Axis

An additional pathway through which mGSH depletion could precipitate coagulation activation operates through the *NLRP3* inflammasome and its downstream executioner, gasdermin D (*GSDMD*). Pyroptotic activation would represent a rapid and self-amplifying coagulation trigger, capable of driving consumptive coagulopathy through a fundamentally different cellular mechanism.

Glutathione peroxidase enzymes within the mitochondrial matrix utilise GSH as their obligate electron donor to neutralise hydrogen peroxide and lipid peroxides. When mGSH is depleted, these enzymes lose their substrates, and mitochondrial ROS accumulates unchecked inside the matrix [33].

Zhou et al. observed that in murine bone marrow-derived macrophages and differentiated THP-1 human monocytic cells, in which mitophagy and autophagy were blocked, transcriptional priming and matrix-derived oxidative stress activated NF-κB. This drove the upregulation of both the *NLRP3* gene and pro-IL-1β, accumulating the dormant molecular components required before the complex can be actively assembled. ROS accumulation was both necessary and sufficient to activate the *NLRP3* inflammasome and drive caspase-1-dependent IL-1β maturation, whilst selective scavenging of mitochondrial ROS suppressed inflammasome activation without affecting general cellular ROS levels, demonstrating that the activating signal was specifically mitochondrial in origin rather than a generalised oxidative stress response. Furthermore, resting *NLRP3* was found to localise to endoplasmic reticulum structures and redistribute to mitochondria and mitochondria-associated membranes upon inflammasome activation, indicating physical proximity between the ROS source and the sensor as a prerequisite for activation [34].

Shimada et al. demonstrated in LPS-primed murine bone marrow-derived macrophages that oxidised mitochondrial DNA released into the cytoplasm during apoptotic mitochondrial dysfunction bound directly to the assembled *NLRP3* complex and triggered caspase-1-dependent IL-1β maturation, with macrophages lacking mtDNA showing severely attenuated inflammasome activation despite ongoing apoptosis. Therefore, oxidised mtDNA could be the obligate activation signal required for *NLRP3*, rather than apoptosis alone [35].

Complementing this, Iyer et al. identified a second, direct activation signal converging on the same inflammasome complex. Cardiolipin, a mitochondria-specific phospholipid normally sequestered within the inner mitochondrial membrane, bound directly to the *NLRP3* protein in a stimulus-dependent manner, as demonstrated by lipid strip binding assays and phospholipid-coated bead pull-downs using recombinant His-tagged human *NLRP3*, with specificity confirmed by the failure of other phospholipids to replicate this interaction. Critically, siRNA-mediated disruption of cardiolipin biosynthesis in THP-1 human monocytic cells inhibited *NLRP3* inflammasome activation across every agonist tested, and the addition of cardiolipin alone to a broken cell system was sufficient to trigger caspase-1 activation [36].

While Iyer et al. demonstrated that this cardiolipin-dependent activation pathway can operate independently of direct mitochondrial ROS generation, this independence highlights the unique gravity of mGSH depletion. Rather than acting as a passive generator of oxidative stress, the collapse of the mGSH pool serves as a dual-action upstream master-switch. Its depletion simultaneously unleashes the mtROS/oxidized mtDNA cascade and drives the catastrophic loss of mitochondrial membrane potential. It is this severe bioenergetic failure that physically forces cardiolipin to flip and externalize to the outer mitochondrial membrane.

In murine bone marrow-derived macrophages and *GSDMD*-deficient mice subjected to bacterial endotoxin challenge, Yang et al. demonstrated that *GSDMD* pore formation drove phosphatidylserine externalisation onto the outer membrane leaflet, which activated tissue factor and initiated the coagulation cascade. In *GSDMD*-deficient animals, coagulation activation and lethality were both prevented, establishing *GSDMD* as a non-redundant executioner of pyroptosis-driven DIC [37]. Concurrently, calcium influx through *GSDMD* pores further amplified tissue factor activation, whilst the release of IL-1β and IL-18 through the same pores propagated the inflammatory signal to neighbouring cells, recruiting additional macrophages and endothelial cells into the pyroptotic cascade. Nrf2 suppression further amplified this entire process by simultaneously removing the transcriptional brake on *NLRP3* gene expression alongside its suppressive effects on GCL subunit transcription, creating a feedforward loop in which enzymatic *GSH* depletion progressively lowered the threshold for inflammasome activation as the septic inflammatory environment intensified. Our deconvolution analysis reveals that the transcriptional suppression of *GCLC* and *SLC25A39* correlates strongly with *NLRP3* upregulation across estimated myeloid populations. Based on this transcriptional convergence, we propose that the relationship between predicted mGSH depletion and *NLRP3* transcript elevation is not merely a generalized consequence of cellular oxidative stress. Rather, it may represent a highly coordinated pathophysiological axis.

#### 3.2.3. NK Cell and Lymphocyte Dysfunction Preventing Sepsis Resolution

GSH depletion may extend its consequences beyond the vascular compartment to impair the immune effector cells required for bacterial clearance, creating the conditions under which sepsis becomes self-perpetuating. This represents a critical link connecting the coagulopathy described to the failure of infection resolution that sustains it.

The GSH-dependence of NK cell cytolytic function was established in vitro by Millman et al. using human peripheral blood NK cells isolated from healthy donors, in which pharmacological GSH depletion using buthionine sulfoximine produced a dose-dependent reduction in *NKG2D* activating receptor surface expression and a corresponding impairment of target cell degranulation capacity, whilst exogenous GSH supplementation restored both NKG2D expression and cytolytic function [38]. The clinical translation of this finding was confirmed by Chiche et al. in a prospective flow cytometry study of 42 ICU sepsis patients and healthy controls, wherein the CD56dimCD16+ NK cell subset, which represents the principal cytolytic effector population responsible for early bacterial and viral clearance, demonstrated significantly reduced degranulation capacity [39].

Critically, in a murine caecal ligation and puncture model, Souza-Fonseca-Guimaraes et al. demonstrated that surviving NK cells following polymicrobial sepsis exhibited cell-intrinsic receptor-mediated functional defects, with Ly49H-mediated degranulation, killing, and effector molecule production all severely reduced on a per-cell basis, DAP12 adaptor protein activation and clustering compromised by TIRF microscopy, and AKT phosphorylation and calcium flux following receptor stimulation diminished. Therefore, the host’s capacity to control secondary cytomegalovirus infection was significantly impaired despite NK cell numbers partially recovering [40], establishing that sepsis-induced NK cell dysfunction creates a specific vulnerability to secondary infection that persists beyond the acute phase.

### 3.3. Central Hypothesis

We propose that multi-point GCL failure, through simultaneous *GCLM* loss, ATP depletion from mitochondrial dysfunction, Nrf2 suppression, and caspase-3 cleavage of *GCLC*, may deplete mGSH in microvascular endothelial cells and could contribute to sepsis-induced DIC through multiple converging mechanisms (Figure 2).

This depletion could further be compounded at the mitochondrial import level by suppression of the *SLC25A39* transporter, which represents the principal carrier responsible for cytoplasmic-to-mitochondrial GSH shuttling. Deconvolution analysis in the pediatric cohort demonstrated downregulation of *SLC25A39* in both monocytes (GSE13904 rho = −0.400) and M1 macrophages (GSE13904 rho = −0.230), whilst *AFG3L2*, the mitochondrial AAA protease that degrades *SLC25A39*, was independently found to be suppressed in both M1 and M2 macrophages of the validation cohort (GSE28750 rho = −0.740 and −0.730, respectively), potentially establishing that mGSH depletion in septic myeloid cells could be driven by impaired import capacity at both the transcriptional and post-translational levels, independently of and in addition to cytoplasmic GSH synthesis failure [10].

Critically, *NFE2L2* transcript abundance positively correlated with monocyte enrichment scores in both cohorts where this association was assessed (GSE13904 rho = +0.473; GSE54514 rho = +0.415), whereas *GCLC* transcripts inversely correlated with monocyte and neutrophil estimates in these same two cohorts (GSE54514 monocytes rho = −0.530; GSE13904 neutrophils rho = −0.340). This divergent pattern suggests that increased *NFE2L2* expression does not correspond to increased *GCLC* transcription in the septic environment, potentially indicating an uncoupling of this compensatory pathway at the transcriptional level [41,42,43].

This framework generates four predictions distinguishable from prior models: (1) GSH/GSSG ratio at ICU admission should independently predict DIC score at 24 h after SOFA adjustment, because we assume GCL failure is mechanistically upstream of DIC rather than merely co-occurring with severity. (2) Plasma GSH should inversely correlate with *NLRP3* pathway markers, specifically IL-18 and *GSDMD* cleavage products, rather than showing a simple severity-dependent depletion pattern. (3) *SLC25A39* and *AFG3L2* expression in circulating monocytes should inversely correlate with mGSH depletion and DIC severity, providing a transcriptionally accessible biomarker of mitochondrial import failure that is independent of cytoplasmic GSH status. (4) IV GSH should reduce both coagulation markers and pyroptosis markers in a manner that NAC, operating upstream of the broken enzyme, cannot replicate, whilst simultaneously restoring *SLC25A39*-dependent mitochondrial import capacity in a way that cytoplasmic cysteine supplementation alone is structurally incapable of achieving.

Studies conducted on murine CD4+ and CD8+ T-cells demonstrated that in lymphocytes carrying a conditional *GCLC* deletion, GSH de novo synthesis was essential for IL-15-mediated metabolic reprogramming and the glutamine catabolism required for effector lymphocyte differentiation. *GCLC*-deficient T-cells failed to undergo mitochondrial biogenesis and oxidative phosphorylation upregulation normally induced by IL-15. Therefore, their differentiation into cytolytic effectors was profoundly impaired despite intact TCR signalling [44].

Sepsis-induced immunosuppression, characterised by impaired NK cytotoxicity, lymphocyte apoptosis, monocyte HLA-DR downregulation, and T-cell exhaustion, is now recognised as the principal driver of late sepsis mortality through susceptibility to secondary bacterial and viral infections, with HSV and CMV reactivation documented in critically ill patients who survived the initial inflammatory phase [45]. Therefore, ongoing bacteraemia, the direct consequence of this immune failure, maintains the LPS-TLR4 signalling that initiated GCL suppression, sustaining *GCLC* and *GCLM* transcriptional repression and perpetuating mGSH depletion in a feedforward cycle that generic antioxidant supplementation cannot interrupt at the enzymatic source [46].

The same GCL failure preventing coagulation resolution simultaneously impairs the NK cell degranulation capacity and T lymphocyte metabolic reprogramming required for bacterial clearance. The resulting bacteraemia maintains the inflammatory stimulus that perpetuates GCL suppression, ensuring that the conditions driving both the coagulopathy and the immune failure are continuously perpetuated.

### 3.4. Articulating the Findings

#### 3.4.1. The Central Bioinformatic Finding

The most mechanistically significant result is the co-occurrence of *GCLC* suppression, *SLC25A39* suppression, and *NLRP3* upregulation in the same myeloid compartment, confirmed across two independent microarray platforms. Specifically, transcript abundances for both GSH synthesis machinery (*GCLC*) and mitochondrial import machinery (*SLC25A39*) were inversely associated with monocyte and M1 macrophage population estimates, the same populations that exhibited the highest positive correlation with *NLRP3* transcripts. We hypothesize that this expression profile reflects a state conducive to unchecked mitochondrial ROS accumulation and *NLRP3* priming, potentially occurring within the cellular populations thought to drive the pyroptotic coagulation arm of DIC. Whether this coordinated transcriptional suppression is causally upstream of *NLRP3* activation via mGSH depletion, or whether these patterns reflect parallel consequences of severe inflammatory activation, requires experimental testing with an isolated in vitro model.

#### 3.4.2. Why Bulk Transcriptomics Alone Is Misleading in Sepsis

Without adjusting for estimated cell-type composition, bulk analysis of a disease characterized by dramatic leukocyte redistribution can produce confounded gene expression interpretations. The apparent *GCLM* upregulation in bulk tissue (log2FC of up to +1.96) might suggest increased compensatory GCL transcription. However, deconvolution analysis reveals that *GCLM* expression does not positively correlate with myeloid enrichment scores, whereas *GCLC* transcript abundance is inversely associated with these same population estimates. Similarly, *NFE2L2* bulk signals are strongly associated with monocyte composition, suggesting that this transcriptional response does not correlate with the expected expression of downstream targets. Any bulk transcriptomic analysis of sepsis without cell-type correction should be interpreted with caution, particularly for genes whose expected expression direction is known.

#### 3.4.3. NK Cell and PRKN Limitations

NK cell numerical depletion was not detected by xCell across the whole-blood datasets (e.g., GSE54514 delta = 0.000, *p*.adj. = 0.776, ns). This likely reflects sensitivity limitations of deconvolution for NK cell detection in the context of dominant myeloid expansion, as well as the fact that NK cell dysfunction in sepsis is primarily functional rather than numerical. Therefore, Section 3.2.3 is the result of direct clinical flow cytometry evidence [39] and in vitro GSH-NK dependency data [38], not the transcriptomic analysis. PRKN (Parkin) was undetectable on all three platforms, preventing assessment of Arm 1 from these datasets.

### 3.5. Why N-Acetylcysteine Has Not Worked

The clinical failure of N-acetylcysteine as a therapeutic intervention in sepsis provides perhaps the most direct evidence that GSH depletion in this context is an enzyme-limited process.

A Cochrane systematic review and meta-analysis of 41 randomised controlled trials was conducted to evaluate the efficacy of N-Acetylcysteine (NAC). This extensive analysis encompassed 2768 adult patients across a highly diverse range of intensive care presentations. The study population included medical patients suffering from acute respiratory distress syndrome (ARDS), multiple organ failure, liver failure, and severe malaria, as well as surgical patients recovering from burns, liver transplantation, and major cardiac, vascular, or abdominal procedures. Despite this robust sample size and broad clinical scope, the authors found that the administration of intravenous NAC yielded no significant clinical benefit over placebo. Critically, NAC therapy failed to reduce overall mortality (RR 1.06, 95% CI 0.79–1.42). Furthermore, it demonstrated no measurable impact on secondary critical outcomes, including ICU length of stay, duration of mechanical ventilation, or the incidence of new-onset organ failure [47].

Methodological heterogeneity was substantial, with NAC dosing ranging from a single intravenous bolus to infusions sustained over seven days, and timing varying widely across trials, representing valid grounds for criticism [38,39]. The fundamental problem may have been represented by the mechanistic formation of GSH. NAC functions exclusively as a cysteine prodrug, premised on the assumption that substrate availability is the rate-limiting determinant of GSH synthesis. This assumption was directly refuted by Lyons et al., in a prospective stable-isotope tracer study on ten septic paediatric patients aged 31 months to 17 years and ten age-matched controls admitted for elective surgery, all receiving a six-hour primed constant intravenous infusion of L-[1-13C]cysteine at a multidisciplinary paediatric ICU. Results showed that plasma cysteine flux was markedly elevated in septic patients compared with controls, confirming that substrate availability was not limiting, yet whole blood GSH synthesis rates were simultaneously and profoundly reduced [19]. Essentially, the substrate was available in excess, but the enzymatic machinery was the failure point.

### 3.6. Therapeutic Strategy

The enzymatic apparatus is proposed to be the failure point; intravenous GSH bypasses this entirely. Pharmacokinetic studies conducted in ten healthy adult volunteers by Aebi et al. demonstrated that following intravenous infusion of 2 g/m^2^ of reduced GSH, plasma total glutathione rose, with near-complete systemic bioavailability, a volume of distribution of 176 ± 107 mL/kg, and a plasma half-life of 14.1 ± 9.2 min, confirming that continuous infusion is required to maintain therapeutic concentrations [48]. This approach has the potential to translate to clinical benefit, as demonstrated by Ortolani et al. in a randomised trial of 30 adult patients admitted to the intensive care units within 24 h of early septic shock diagnosis. Patients were allocated to one of three groups. The first received standard septic shock therapy alone. The second received standard therapy plus intravenous GSH administered at 70 mg/kg/day. The third received standard therapy plus intravenous GSH at the same dose combined with intravenous N-acetylcysteine (NAC) at 75 mg/kg/day, both delivered by continuous infusion. The rationale for the combined group reflects the biosynthetic logic established earlier. NAC serves as a cysteine donor supporting whatever residual de novo GSH synthesis capacity remains, whilst exogenous GSH supplies the finished antioxidant product independently of enzymatic conversion, allowing the trial to distinguish substrate support from direct repletion.

To capture the functional consequences of this intervention, the authors assessed peroxidative damage using a multi-modal panel. Expired ethane, a volatile marker of lipid peroxidation, was measured. Plasma malondialdehyde—a stable aldehyde end-product of lipid peroxidation—and erythrocyte deformability—assessed because red cell membrane integrity is highly sensitive to oxidative damage and directly influences microvascular flow—served as functional correlates linking oxidative stress to the microcirculatory dysfunction characteristic of septic shock. Complement activation was included as a marker of the broader inflammatory response accompanying oxidative injury. These measures were recorded at admission to establish baseline severity, and again on days 3 and 5 to track the trajectory of treatment response.

By day 5, both GSH-treated groups showed significant reductions in peroxidative indices relative to both the control group and each group’s own baseline values, indicating that exogenous glutathione was not only biologically active but capable of measurably reversing oxidative injury despite the ongoing septic environment. The combined GSH-NAC group produced further and more rapid reductions than GSH alone, consistent with an additive or synergistic effect of pairing substrate support with direct product repletion. This biochemical improvement was accompanied by significant improvements in clinical severity scores.

These findings establish that intravenous GSH, at a dose of 70 mg/kg/day, was both safe and biologically active within the septic shock environment, and that its combination with NAC yielded the most rapid biochemical and clinical improvement [49].

However, our cross-cohort analysis indicates that cytoplasmic repletion alone may not resolve the hypothesized mitochondrial deficit, given the concurrent transcriptional suppression of both *SLC25A39* and *AFG3L2*. To address this mitochondrial import trap, future experimental models might investigate the concurrent use of small-molecule iron ionophores, such as hinokitiol. Hinokitiol acts as a biomimetic iron chaperone, theoretically driving the biogenesis of the iron–sulfur clusters required to shield *SLC25A39* from proteolytic cleavage [10] (Figure 3).

The existing toxicology literature on hinokitiol is entirely preclinical and predominantly non-parenteral, and several findings raise specific concerns for the septic shock setting proposed here. Acute toxicity studies report an intraperitoneal LD50 of 191 mg/kg and an oral LD50 of 760 mg/kg in mice [50]; a 13-week dietary study in F344 rats found dose-dependent hepatic, haematological, and body-weight effects at higher intakes; and myocarditis has been separately reported in male F344 rats on hinokitiol-containing diets [51]. A recent review of hinokitiol’s anticancer pharmacology explicitly notes the absence of human clinical trials, limited pharmacokinetic and pharmacodynamic data, and insufficient toxicity profiling, alongside context-dependent effects on ferroptosis that can run in either direction depending on dose and cellular context [52]. At higher concentrations, hinokitiol–Fe(III) complexes aggregate and lose transmembrane iron-mobilising capacity, meaning a simple dose-escalation strategy cannot be assumed to produce a proportionate increase in the intended effect [53]. This risk is plausibly amplified, rather than mitigated, by the septic environment itself. Sepsis is independently characterised by hepcidin-driven trapping of iron intracellularly (including in mitochondria), hyperferritinemia, and depressed GPX4 activity, and ferroptosis is an established contributor to sepsis-associated lung, kidney, liver, and cardiac injury [54,55]. Given these gaps, hinokitiol should be regarded as mechanistically motivated but not yet ready for the human dose-escalation trial outlined in Section 3.9, and a dedicated safety programme should be implemented.

A critical constraint governing the dosing of this combined strategy is the ceiling imposed by reductive stress. Cao et al., using isolated bovine heart mitochondria, demonstrated that supraphysiological GSH concentrations impair mitochondrial electron transport chain function by over-reducing the thioredoxin-2 and peroxiredoxin-III system, establishing that excess reducing equivalents are themselves damaging to mitochondrial respiration [56]. The pharmacodynamic target would therefore be the restoration of a physiological GSH/GSSG ratio (100:1) rather than the maximization of absolute GSH concentration.

For the post-ICU recovery phase, where immune deficits may persist, oral liposomal GSH formulations have shown preliminary efficacy in elevating intracellular GSH and recovering NK cell cytotoxicity in healthy volunteers.

The combination of these compounds for therapeutic administration has not been studied in humans and requires experimental validation.

Sinha et al. addressed this using a liposomal GSH formulation, designed to protect the tripeptide from enzymatic breakdown and enhance cellular uptake. In a one-month randomised double-blind placebo-controlled pilot trial conducted in 12 healthy adults, participants received liposomal GSH at one of two doses, 500 mg/day or 1000 mg/day, allowing the authors to assess whether the immune effects observed were dose-dependent. Because the trial was conducted in healthy volunteers rather than septic patients, it provided a controlled setting in which to establish the relationship between GSH repletion and immune function without the confounding influence of ongoing infection or multi-organ dysfunction.

Supplementation produced measurable increases in GSH content across four compartments. Whole blood, erythrocyte, plasma, and peripheral blood mononuclear cell (PBMC) intracellular GSH levels all rose within one week of treatment initiation, indicating that the liposomal formulation successfully overcame the bioavailability limitations of earlier oral GSH approaches. Concurrently, urinary oxidative stress biomarkers declined, providing independent biochemical confirmation that the elevated GSH pool was functionally active in neutralising oxidative burden rather than simply accumulating without effect.

The immune consequences of this repletion closely tracked the observed changes in cellular GSH content. By week two, NK cell cytotoxicity had increased by up to 400%, a magnitude of recovery consistent with the GSH-dependence of *NKG2D* receptor expression and degranulation capacity established earlier in Section 3.2.3. In the high-dose group, lymphocyte proliferation increased by 60%, suggesting that the metabolic reprogramming required for effector lymphocyte differentiation, previously shown to depend on intact *GCLC*-mediated GSH synthesis, could similarly be supported through direct GSH provision. Critically, the time course of these immune improvements coincided precisely with the rise in PBMC intracellular GSH content [57].

High-dose vitamin C, NAC, and broad antioxidant strategies have consistently failed in the septic shock setting, most recently in the CITRIS-ALI randomised controlled trial of intravenous vitamin C in 167 adult ICU patients with sepsis-associated ARDS, which demonstrated no significant effect on organ failure scores or 28-day mortality [58]. All of these interventions attempt to reduce oxidative stress non-specifically, without restoring the enzymatic machinery responsible for endogenous antioxidant production, and none addresses the protease-importer bottleneck that prevents mitochondrial GSH restoration even when cytoplasmic substrate is available.

### 3.7. Preliminary Taxonomy of DIC Subtypes by Predicted GSH Axis Relevance

The three-arm framework predicts GSH-axis involvement should be greatest where endothelial oxidative destruction, *NLRP3* pyroptosis, and immune dysfunction are primary drivers. The following taxonomy is explicitly theoretical and generates testable predictions for Experiment 5; it does not summarise established evidence (Table 4).

### 3.8. Limitations

Within this paper, there are limitations which require acknowledgement.

#### 3.8.1. No Direct mGSH Measurement

Whole-blood transcriptomics cannot assess mGSH levels in microvascular endothelial cells, the proposed primary target of GCL transcriptional suppression. While the *GCLC* and *SLC25A39* suppression signals strongly correlate with circulating myeloid cell estimates (relevant to Arms 2 and 3), these bulk data do not directly test the endothelial Arm 1 prediction. Experiment 1 (in vitro endothelial model) is the minimum requirement to address this gap.

#### 3.8.2. Observational Design

Spearman correlations establish co-occurrence, not causality. Whether myeloid *GCLC/SLC25A39* suppression is causally upstream of *NLRP3* activation through mGSH depletion, or both reflect parallel consequences of myeloid activation, cannot be resolved from cross-sectional transcriptomics. The three-level myeloid convergence is consistent with the hypothesis but does not prove it.

#### 3.8.3. PRKN Undetectable

Parkin analysis data were insufficient across all three platforms. The *PINK1/Parkin* mitophagic failure arm (Arm 1) cannot be assessed from these datasets and rests entirely on the mechanistic literature [59,60,61]. Endothelial cell-specific assessment of Parkin oxidative inactivation is the target of Experiment 1b.

#### 3.8.4. NK Cell Depletion Not Detected

xCell detected no significant NK cell score change in any dataset. Arm 3 is supported by direct clinical evidence [39] and in vitro GSH-NK dependency data [38] rather than these transcriptomic analyses. The deconvolution result is consistent with functional rather than numerical NK cell impairment in sepsis.

#### 3.8.5. GSE28750 Dataset Is Functionally Underpowered

With only 10 sepsis patients, GSE28750 cannot generate reliable deconvolution estimates or significant DE results. Its contribution throughout is directional confirmation only. The primary statistical weight rests on GSE13904 (n = 117) and GSE54514 (n = 53).

#### 3.8.6. No DIC Stratification

The four GEO cohorts compare sepsis/septic shock against healthy controls; none report ISTH DIC score, coagulation parameters, or fibrinogen/platelet trajectories. Consequently, the present bioinformatic findings support a hypothesis about GSH-axis dysfunction in sepsis broadly. Their extension to DIC as a specific mechanism rests on the independent mechanistic literature reviewed in Section 3.2, Section 3.3, Section 3.4, Section 3.5, Section 3.6 and Section 3.7 and requires the DIC-stratified prospective cohort, proposed in Section 3.9 (Experiment 1) for direct evidence.

#### 3.8.7. Gene–Cell-Type Associations

xCell provides computational estimates of cell-type enrichment from bulk expression signatures, not flow-sorted or single-cell ground truth. Gene–cell-type associations identified via Spearman’s correlation are consistent with targeted transcriptional regulation within these compartments. However, they could also reflect residual estimation errors in the enrichment scores or activation-state-dependent shifts in the marker genes that xCell itself relies on. Direct confirmation would require flow cytometry- or scRNA-seq-sorted populations.

#### 3.8.8. Protein Abundance Cannot Be Measured Directly

*GCLC* and *GCLM* transcript abundance is used throughout as a proxy for GCL pathway activity. The mRNA level does not directly measure GCL protein abundance, holoenzyme assembly, or catalytic activity, each of which can be regulated independently of transcription (as shown by the caspase-3 cleavage and Fe–S cofactor mechanisms discussed in Section 3.1). The enzymatic-activity claims in Section 3.1 rest on the cited biochemical literature, not on the transcriptomic data generated in this paper; direct enzymatic validation is proposed in Section 3.9.

#### 3.8.9. Dataset Heterogeneity

GSE13904 (Wong et al.) profiles a pediatric SIRS/sepsis/septic-shock cohort, although we did not include any SIRS samples present within this dataset. GSE54514 and GSE28750 profile adult populations. This age heterogeneity limits cross-dataset generalisability; consequently, cross-platform directional consistency, rather than pooled effect size, is emphasised throughout this study.

### 3.9. Experimental Studies That Can Be Conducted Based on Bioinformatic Findings

To establish the bioinformatic findings, we propose a series of experiments aimed at validating our hypothesis.

The most immediately actionable and highest-priority study is a prospective observational cohort in which plasma and PBMC intracellular GSH are measured at ICU admission before any antioxidant administration has been given. The central question this study addresses is whether admission GSH independently predicts overt DIC score at 24 h after adjustment for SOFA score, tested by multivariate logistic regression; if GCL failure is mechanistically upstream of DIC rather than merely co-occurring with severity, this relationship should hold independently of illness severity. Branching experiments may extend this to 28-day mortality via the GSH/GSSG ratio, serial GSH trajectory over the admission period, and correlation with IL-18 and *GSDMD* cleavage products as markers of pyroptotic activation.

This was modelled in vitro using two complementary endothelial systems: HUVECs, representing large-vessel endothelium, and HMEC-1 cells, representing the microvascular compartment preferentially targeted in DIC, both being exposed simultaneously to plasma from patients with overt DIC, SOFA-matched non-DIC septic shock, and healthy controls, allowing the endothelial response to be attributed to DIC-specific plasma constituents rather than generalised severity. Primary endpoints focus on the mitochondrial and import layer of the proposed mechanism: mitochondrial membrane potential measured by JC-1, mGSH quantified by ThiolTracker Violet, and *SLC25A39* protein abundance. Parallel wells receiving physiological-dose exogenous GSH constitute the intervention arm, directly testing whether restoration of GSH availability is sufficient to rescue the endothelial phenotype. A mechanistic extension of this experiment then characterises the full pathway in detail, including *PINK1* accumulation, Parkin activity, *NLRP3* transcript upregulation markers, tissue factor expression, and caspase-3 cleavage, allowing each section of the proposed DIC mechanism to be confirmed or refuted at the cellular level.

In vivo validation came from the CLP model, in which polymicrobial peritonitis is induced surgically to reproduce the phases of human sepsis [62], and was used to compare intravenous GSH directly against NAC and saline as comparators. Primary endpoints mirror the clinical study, allowing the head-to-head comparison between GSH and NAC that the clinical literature currently lacks: DIC score, endothelial mGSH, *NLRP3* upregulation markers, and NK cell function. This experiment requires an animal facility and an institutional principal investigator holding a personal animal licence.

Once the mechanistic and observational evidence base is established, the clinical scope is extended by enrolling cancer-associated, obstetric, and genetic thrombophilia DIC subtypes alongside the septic cohort. Genetic thrombophilia is included deliberately as a predicted low-relevance internal control, on the basis that its DIC mechanism is coagulation-intrinsic rather than GCL-driven; a null result in this subgroup would strengthen the specificity of any positive finding in the septic and obstetric arms whilst requiring minimal additional cost.

The programme concludes, on a five-to-seven-year horizon and contingent on convergent positive results from the preceding studies, with a dose-escalation intravenous GSH trial in septic shock, using the GSH/GSSG ratio as the primary pharmacodynamic endpoint to define the dose required to restore redox homeostasis at the tissue level before proceeding to efficacy endpoints.

## 4. Materials and Methods

### 4.1. Dataset Selection

Two independent adult and one pediatric whole-blood GEO datasets were selected for transcriptomic analysis on the basis of biological relevance, platform diversity, and sample size. GSE54514, generated by Parnell et al. from whole blood drawn from 35 septic shock patients with pneumonia as the primary infectious source and 18 healthy controls profiled on the Illumina HumanHT-12 v4 platform, provided the largest homogeneous severity stratum [41]. GSE13904, generated by Wong et al., originally profiled pediatric patients across the clinical continuum of uncomplicated sepsis, severe sepsis, and septic shock, as well as patients with non-infectious systemic inflammatory response syndrome (SIRS). To strictly isolate the infectious pathophysiology, all SIRS subjects were explicitly excluded from our analysis. This yielded a final analytical cohort of 99 pediatric sepsis patients, including both survivors and non-survivors, alongside 18 healthy controls profiled on the Affymetrix U133 Plus 2.0 platform, allowing expression profiles to be examined across a clinically heterogeneous infectious population [42]. GSE28750, generated by Sutherland et al. from 10 adult ICU sepsis patients and 20 healthy controls on the Affymetrix U133 Plus 2.0 platform, provided an independent validation cohort on the same array architecture as GSE13904, permitting cross-dataset consistency to be distinguished from platform-specific artefact [43]. The total transcriptomic cohort comprised 144 sepsis patients and 56 controls across two independent microarray platforms (Table 5).

### 4.2. Longitudinal Validation Cohort (Cell-Specific Temporal Profiling)

To resolve the kinetic and cell-intrinsic properties of the identified pathways beyond what whole-blood bulk transcriptomics could provide. GSE46955—generated by Shalova et al. [63] using the Illumina HumanRef-8 v2.0 expression BeadChip (GPL6104) on isolated peripheral blood monocytes collected from eight patients during acute Gram-negative sepsis and matched longitudinally following clinical recovery alongside six healthy donor controls, comprising 22 samples in total across acute, recovery, and control timepoints—provided the only available dataset combining cell-type specificity, temporal resolution, and transcript-level quantification in the same individuals.

### 4.3. Computational Methodology

Gene targets were selected based on the mechanistic framework described in Section 3.1, Section 3.2 and Section 3.3, comprising the GCL biosynthetic subunits *GCLC* and *GCLM*, the Nrf2 transcription factor encoded by *NFE2L2*, the pyroptosis executioner axis *NLRP3* and *GSDMD*, the mitophagy initiator *PINK1*, the receptor-mediated mitophagy targets *BNIP3L* and *FUNDC1*, and the mitochondrial GSH transporter family members *SLC25A39*, *SLC25A40*, and *AFG3L2*. *PRKN*, encoding the mitophagy E3 ligase Parkin, was examined, but the data provided across all three transcriptomic platforms were insufficient and were therefore excluded from the analysis. All three cohorts were restricted to the day 1 baseline sample per patient prior to all downstream testing, preventing pseudoreplication from repeated within-patient sampling. Differential expression between sepsis patients and controls was then assessed using the Mann–Whitney U test with Benjamini–Hochberg false discovery rate correction applied across all tested genes [64], with significance thresholds reported as *p*.adj < 0.001 (***), <0.01 (**), and <0.05 (*). This non-parametric approach was selected, given the non-normal distribution of expression values and the variable sample sizes across cohorts, and its robustness to outliers was considered particularly important given the clinical heterogeneity of the sepsis populations.

To mitigate platform-specific bias between the Illumina (GSE54514) and Affymetrix (GSE13904, GSE28750) arrays, all statistical analyses were restricted to within-dataset comparisons. Neither raw expression values nor xCell enrichment scores were pooled. We utilized Spearman’s rank correlation, which is inherently invariant to monotonic differences in platform dynamic range, thereby ensuring correlations were independent of underlying intensity scales. This was empirically validated via z-score transformation (maximum |Δrho| < 1 × 10^−6^). Furthermore, standard cross-platform batch correction algorithms (e.g., ComBat) were omitted. Because dataset identity is entirely confounded with distinct patient cohorts, global batch correction risks suppressing true biological variance. Consequently, a conservative, strictly within-platform, rank-based analytical approach was utilized throughout.

To account for apparent expression shifts attributable to alterations in circulating cell-type composition, a critical confound in whole-blood sepsis transcriptomics given the well-documented leukocyte redistribution that accompanies systemic infection, xCell computational deconvolution [65] was applied to estimate relative enrichment scores across 64 immune and stromal cell types from the bulk expression profiles of each sample. Spearman rank correlations between bulk gene expression values and cell-type enrichment scores were then computed with Benjamini–Hochberg correction. Genes whose expression correlated strongly with a specific cell-type score were classified as compositionally associated, indicating that their differential expression in the bulk transcriptome likely tracks with the relative enrichment or depletion of that specific leukocyte population.

Because GSE46955 contains repeated acute and recovery-phase samples from the same patients, the relationship between *AFG3L2* and *SLC25A39* transcript abundance in this dataset was modeled separately from the day 1, single-time-point cohorts described above. Raw expression data were retrieved directly from GEO, and probes mapping to *AFG3L2* or *SLC25A39* were collapsed to a single value per gene per sample by taking the maximum expression across probes. LPS-stimulated samples were excluded, retaining only the 22 basal (unstimulated) samples across acute, recovery, and healthy control time points from 14 individuals. A linear mixed-effects model was then fit with *SLC25A39* as the outcome, *AFG3L2* as a fixed effect, and a random intercept per patient to account for the non-independence of repeated within-patient measurements.

All data manipulation, visualization, and statistical analyses were performed using R software (version 4.5.2, R Foundation for Statistical Computing, Vienna, Austria) running on Windows 10 (Microsoft Corporation, Redmond, WA, USA), and RStudio (version 2026.01.1, Posit Software, PBC, Boston, MA, USA). Specific packages utilized included the tidyverse suite (version 2.0.0), ggplot2 (version 4.0.2), ggpubr (version 0.6.3), pheatmap (version 1.0.13), RColorBrewer (version 1.1.3), rstatix (version 0.7.3), lmerTest (version 3.2.1), and writexl (version 1.5.4). Gene expression data was accessed and processed utilizing the Bioconductor packages GEOquery (version 2.78.0), Biobase (version 2.70.0), limma (version 3.66.0), and xCell (version 1.1.0). The complete R scripts used for data extraction, strict day 1 deduplication, bulk signal differential expression, xCell deconvolution, and cell population shift analysis are publicly available on GitHub (https://github.com/felixbadiu9/The-enzymatic-mitochondrial-import-bottleneck-in-Sepsis, acceesed on 28 July 2026).

## 5. Conclusions

Sepsis has resisted therapeutic intervention for decades because the field has continuously addressed its downstream consequences, instead of its mechanistic root. The framework presented here reframes the origin of this coagulopathy around a two-stage collapse of the mitochondrial glutathione (mGSH) axis within the myeloid and endothelial compartments. The systemic collapse may be enzyme- and transport-limited, not substrate-limited. This key distinction explains why the persistent clinical use of N-acetylcysteine (NAC) and other antioxidant substrates remains largely ineffective. The acute pathology operates through concurrent mechanisms: a severe loss of GCL synthetic capacity—via *GCLM* loss, ATP depletion, Nrf2 transcriptional uncoupling, and caspase-3-mediated cleavage—paired with the suppression of the mGSH importer, *SLC25A39*. Based on our transcriptomic findings of mGSH import machinery suppression, we hypothesize that the downstream consequences may propagate through multiple convergent axes described in the existing literature. These potentially include *PINK1/Parkin*-driven procoagulant endothelial apoptosis, *NLRP3*/*GSDMD* pyroptotic coagulation, and sustained lymphocyte dysfunction.

The bioinformatic analysis presented here, spanning sepsis patients across three whole-blood transcriptomic cohorts alongside a longitudinal monocyte transcriptomic dataset, provides compelling evidence that the transcriptional suppression of GSH synthesis and mitochondrial import machinery co-occurs with inflammasome-associated transcriptional changes in myeloid cells during sepsis. Deconvolution analysis demonstrated that *GCLC* transcript abundance is inversely associated with the estimated enrichment of the same myeloid populations that correlate positively with *NLRP3* transcription.

Crucially, in the isolated monocyte cohort, a linear mixed-effects model accounting for repeated within-patient sampling showed that *AFG3L2* and *SLC25A39* are significantly co-regulated at the transcript level within individual patients (β = 0.404, *p* = 0.0122). Because the septic environment is known to compromise the iron–sulfur (Fe–S) clusters required to stabilize *SLC25A39*, we hypothesize that this tight coupling may reflect a state in which newly synthesized transporters remain vulnerable to proteolytic cleavage. This multi-level transcriptional picture, comprising acute suppression of cytosolic synthesis machinery alongside a tightly co-regulated importer/protease axis, has not been previously described and represents the central hypothesis-generating contribution of this paper. The therapeutic implication is highly specific, testable, and demands a synchronized, dual-hit intervention. We hypothesize that bypassing the broken enzymatic machinery through intravenous glutathione combined with an Fe–S cluster-promoting agent, such as hinokitiol, could mechanistically address these findings. However, this remains strictly a preclinical hypothesis that requires extensive investigation to resolve existing safety concerns, particularly the potential for ionophore toxicity in humans. This combined rationale addresses the failure at both the cytoplasmic synthesis and mitochondrial import levels simultaneously, a dual targeting that no existing intervention achieves. The experimental programme proposed in Section 3.9 is designed to rigorously test the falsifiable predictions that distinguish this framework. The ultimate ambition of this work is to rationally dismantle the enzymatic and transport source of coagulopathy for the first time.

## Figures and Tables

**Figure 1:**
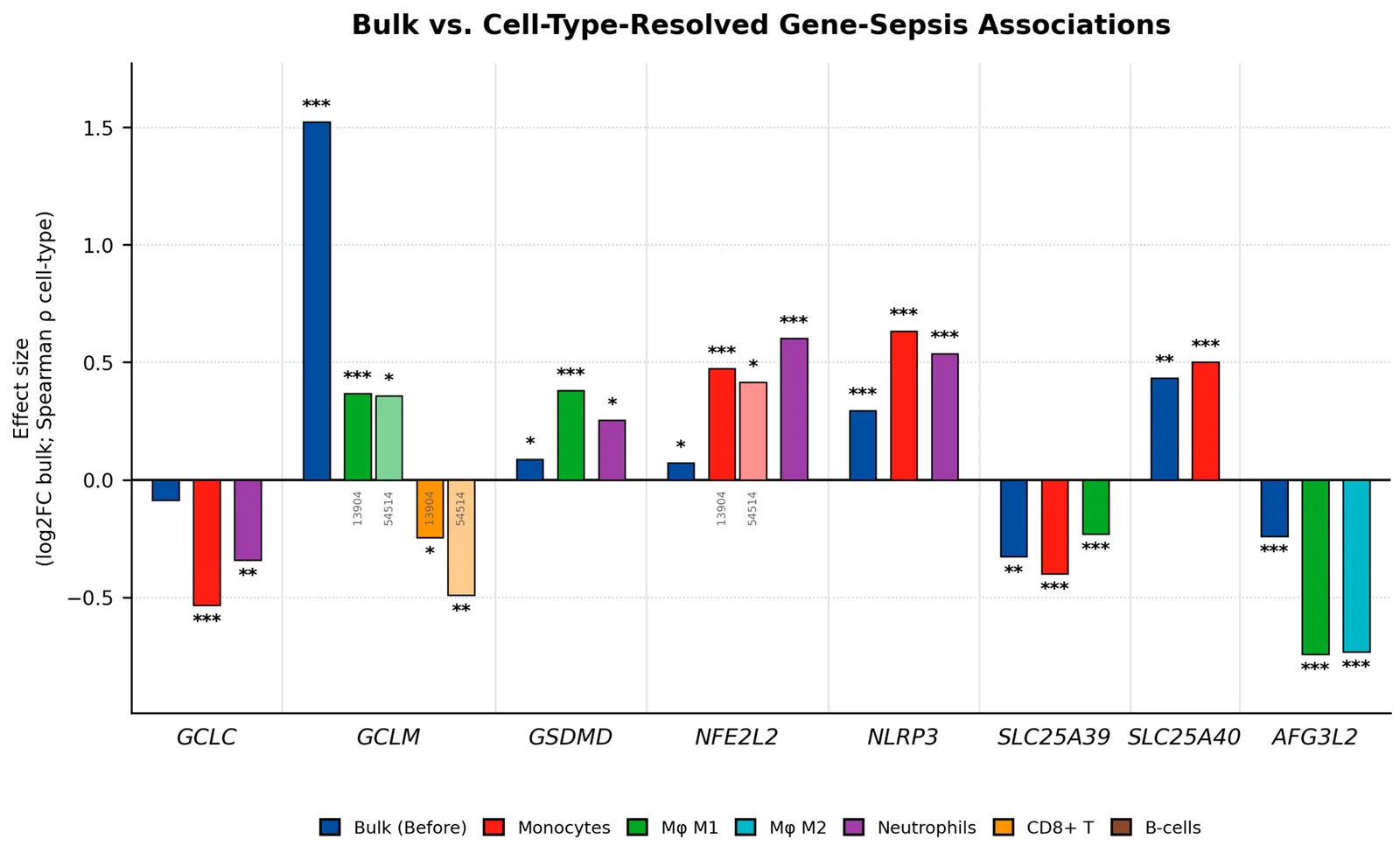
Bulk transcript-level versus cell-type-resolved gene–sepsis associations across mitochondrial-glutathione and inflammasome-pathway genes. Blue bars show bulk differential expression (log2 fold-change, sepsis vs. healthy control) in GSE13904 day 1 samples, Benjamini–Hochberg-adjusted. Colored bars show cell-type-resolved associations (Spearman’s ρ between gene expression and xCell-estimated cell-type enrichment score, BH-adjusted) for monocytes, M1 macrophages, M2 macrophages, neutrophils, CD8+ T-cells, and B-cells. Because log2FC and Spearman’s ρ are different statistics, values are plotted on a shared axis for sign and significance comparison only; bar heights are not directly comparable between the two categories. Where two independent datasets reported an association for the same gene/cell-type pair, both are shown side by side, with the second dataset displayed as a lighter shade of the same color. Small vertical labels beneath these bars give the GEO accession suffix (e.g., “13904” = GSE13904, “54514” = GSE54514). *AFG3L2* cell-type-resolved values are derived from GSE28750, the only dataset providing monocyte/macrophage-resolved data for this gene; its bulk value is from GSE13904, as for all other genes. *PINK1* and *BNIP3L* are omitted. Asterisks denote BH-adjusted significance: * *p*.adj. < 0.05; ** *p*.adj. < 0.01; *** *p*.adj. < 0.001. Bars without asterisks were not statistically significant (ns). Graphs were created with Python 3.12 by Badiu, F.

**Figure 2:**
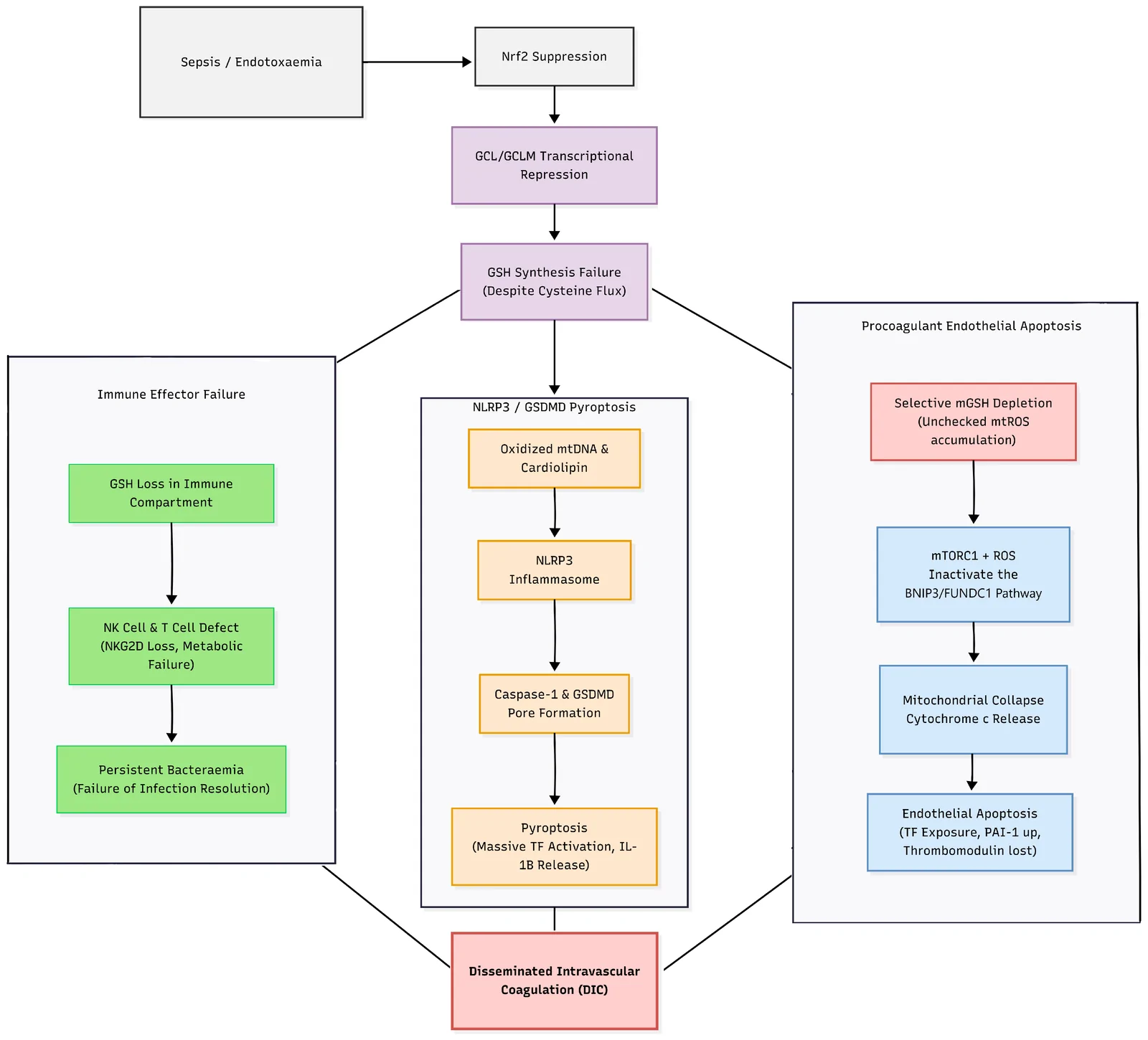
Proposed mechanistic pathway linking sepsis-induced Nrf2 suppression and glutathione synthesis failure to disseminated intravascular coagulation (DIC). The flowchart illustrates the hypothesized cascade from initial systemic triggers to the terminal event of DIC, separated into distinct color-coded mechanistic pathways. Immune Effector Failure (Green): Driven by lymphoid GSH depletion, this pathway induces NK and T-cell defects (characterized by NKG2D loss and metabolic failure), potentially preventing infection resolution and driving persistent bacteremia. Inflammatory Cell Death (Yellow/Orange): This traces the pyroptosis pathway from mitochondrial danger signals (oxidized mtDNA and cardiolipin) to *NLRP3* inflammasome assembly and caspase-1 and *GSDMD* pore formation, culminating in massive IL-1B activation and IL-18 release. Vascular Endothelial Collapse (Blue): This details the steps leading to procoagulant endothelial apoptosis. Critical Tipping Point and Terminal Event (Red): Selective mGSH depletion and DIC are highlighted in red to denote the hypothesized causal link between the critical intracellular redox tipping point and the terminal event of disseminated intravascular coagulation (DIC). Created with Mermaid.js by Badiu, F.

**Figure 3:**
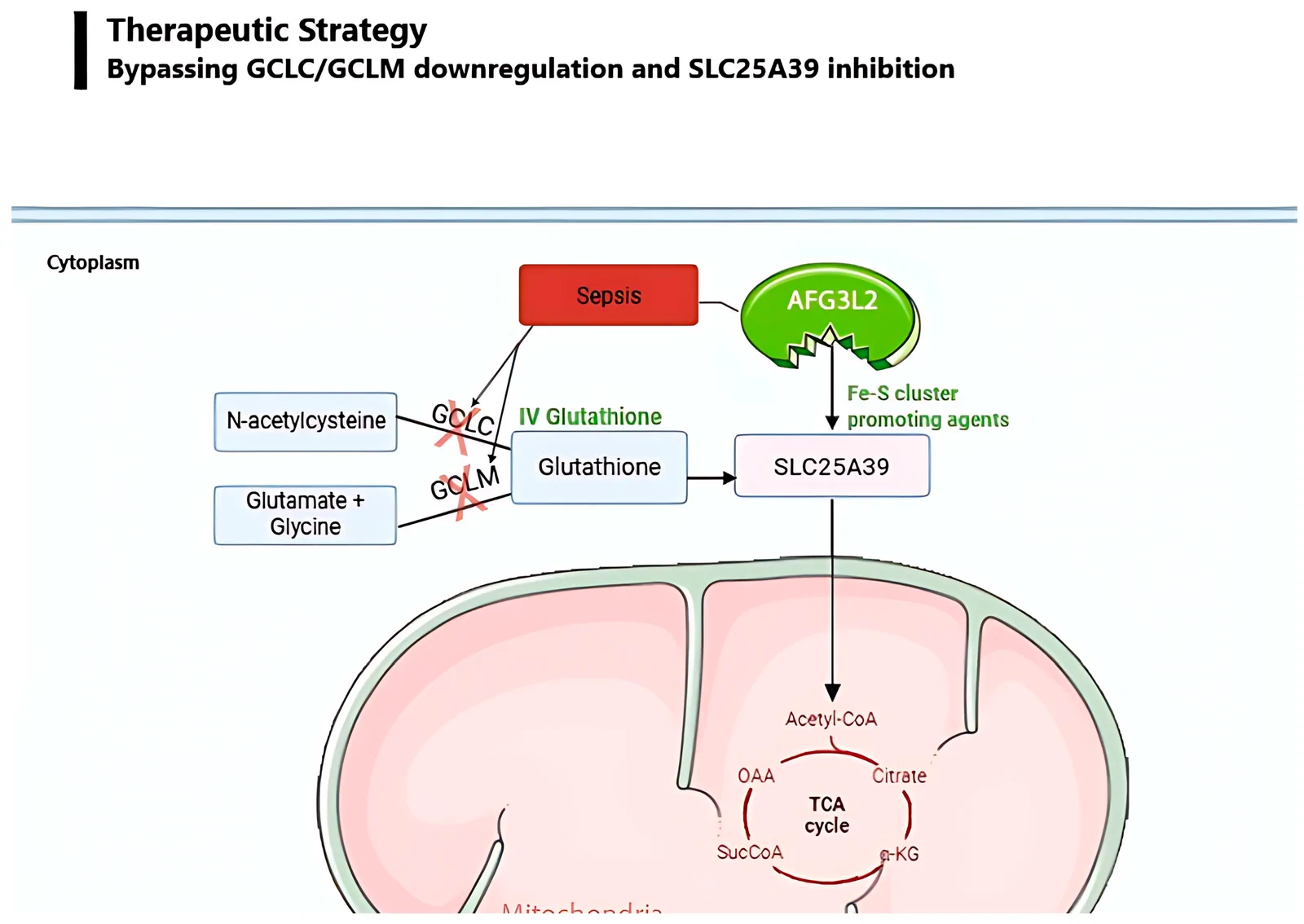
This schematic illustrates a dual-intervention approach designed to rescue glutathione synthesis and mitochondrial import during sepsis. Sepsis induces the functional downregulation of the enzymes *GCLC* and *GCLM*, effectively blocking the de novo synthesis of cytoplasmic glutathione from precursor molecules (N-acetylcysteine, glutamate, and glycine). To bypass this primary biosynthetic blockade, direct administration of IV glutathione is proposed to restore the cytoplasmic pool. Downstream, sepsis-associated pathways involving the *AFG3L2* protease lead to the inhibition of *SLC25A39*, the critical mitochondrial glutathione transporter. A secondary intervention utilizing Fe–S cluster-promoting agents is proposed to bypass or rescue this *SLC25A39* inhibition, thereby restoring essential glutathione import into the mitochondrial matrix to protect and support core metabolic processes, such as the TCA cycle. Enzyme and mitochondria illustrations were adapted from Servier Medical Art (https://smart.servier.com), licensed under CC BY 4.0 by Badiu, F.

**Table 1:** Bioinformatic analysis of relevant targets: cross-dataset differential expression of glutathione-synthesis, mitochondrial-transport, and inflammasome-pathway genes across three independent sepsis cohorts (sepsis vs. control). Log2FC = log2 fold-change; *p*.adj = Benjamini–Hochberg corrected *p*-value. Statistical significance is indicated by asterisks: ns = not-significant, (*p* > 0.05); * (*p* ≤ 0.05); ** (*p* ≤ 0.01); *** (*p* ≤ 0.001).

Dataset	Gene	Log2FC	*p*.adj.	Sig.
GSE13904	*AFG3L2*	−0.240	<0.0001	***
GSE28750	*AFG3L2*	−0.564	0.00059	***
GSE54514	*AFG3L2*	+0.079	0.325	ns
GSE13904	*BNIP3L*	−0.145	0.215	ns
GSE28750	*BNIP3L*	+0.916	0.00109	**
GSE54514	*BNIP3L*	−0.261	0.184	ns
GSE13904	*FUNDC1*	−0.525	0.000186	***
GSE28750	*FUNDC1*	−1.150	0.000146	***
GSE54514	*FUNDC1*	−0.154	0.0250	*
GSE13904	*GCLC*	−0.088	0.675	ns
GSE28750	*GCLC*	+0.101	0.230	ns
GSE54514	*GCLC*	−0.141	0.408	ns
GSE13904	*GCLM*	+1.522	<0.0001	***
GSE28750	*GCLM*	+1.964	0.0004	***
GSE54514	*GCLM*	−0.136	0.408	ns
GSE13904	*GSDMD*	+0.087	0.048	*
GSE54514	*GSDMD*	−0.077	0.884	ns
GSE13904	*NFE2L2*	+0.071	0.048	*
GSE54514	*NFE2L2*	−0.282	0.171	ns
GSE13904	*NLRP3*	+0.295	<0.0001	***
GSE28750	*NLRP3*	+0.277	0.211	ns
GSE54514	*NLRP3*	−0.063	0.408	ns
GSE13904	*PINK1*	+0.272	0.048	*
GSE54514	*PINK1*	−0.271	0.171	ns
GSE13904	*SLC25A39*	−0.327	0.0071	**
GSE28750	*SLC25A39*	−0.121	0.775	ns
GSE54514	*SLC25A39*	−0.149	0.498	ns
GSE13904	*SLC25A40*	+0.434	0.0021	**
GSE28750	*SLC25A40*	+1.595	0.0019	**
GSE54514	*SLC25A40*	+0.137	0.993	ns

**Table 2:** Sepsis-induced shifts in estimated immune cell composition. Cell-type relative abundance scores were estimated from bulk whole-blood microarrays using the xCell deconvolution algorithm. To ensure rigorous statistical independence, all longitudinal cohorts (GSE54514, GSE13904, and GSE28750) were strictly filtered to retain only the day 1 baseline sample per unique patient, preventing pseudoreplication. Differences in estimated cell-type fractions between sepsis and healthy control patients were evaluated using the non-parametric Wilcoxon rank-sum test. The delta (Δ) score represents the difference between the median sepsis composition score and the median control composition score. *p*-values were adjusted for multiple comparisons within each dataset using the Benjamini–Hochberg (BH) false discovery rate method. Statistical significance is indicated by asterisks: ns = not significant (*p* > 0.05); ** (*p* ≤ 0.01); *** (*p* ≤ 0.001).

Dataset	Method	Cell Type	Δ (Composition Score)	*p*.adj.	Sig.	Direction
GSE54514	Wilcoxon median Δ	Monocytes	−0.004	0.963	ns	Lower
GSE54514	Wilcoxon median Δ	Neutrophils	−0.0003	0.776	ns	Lower
GSE54514	Wilcoxon median Δ	NK cells	0.000	0.776	ns	-
GSE54514	Wilcoxon median Δ	CD8+ T-cells	−0.017	0.864	ns	Lower
GSE54514	Wilcoxon median Δ	CD4+ T-cells	0.000	0.754	ns	-
GSE54514	Wilcoxon median Δ	B-cells	+0.014	0.497	ns	Higher
GSE54514	Wilcoxon median Δ	Macrophages M1	0.000	0.424	ns	-
GSE54514	Wilcoxon median Δ	Macrophages M2	0.000	0.689	ns	-
GSE13904	Wilcoxon median Δ	Monocytes	+0.413	4.20 × 10^−6^	***	Higher
GSE13904	Wilcoxon median Δ	Neutrophils	+0.460	4.20 × 10^−6^	***	Higher
GSE13904	Wilcoxon median Δ	CD8+ T-cells	−0.377	4.20 × 10^−6^	***	Lower
GSE13904	Wilcoxon median Δ	CD4+ T-cells	−0.076	0.00074	***	Lower
GSE13904	Wilcoxon median Δ	B-cells	−0.601	5.05 × 10^−5^	***	Lower
GSE13904	Wilcoxon median Δ	Macrophages M1	+0.079	7.68 × 10^−6^	***	Higher
GSE13904	Wilcoxon median Δ	Macrophages M2	+0.057	6.12 × 10^−6^	***	Higher
GSE28750	Wilcoxon median Δ	CD8+ T-cells	−0.156	4.70 × 10^−5^	***	Lower
GSE28750	Wilcoxon median Δ	CD4+ T-cells	−0.017	0.00156	**	Lower
GSE28750	Wilcoxon median Δ	B-cells	−0.092	4.77 × 10^−5^	***	Lower
GSE28750	Wilcoxon median Δ	Macrophages M1	+0.012	4.70 × 10^−5^	***	Higher
GSE28750	Wilcoxon median Δ	Macrophages M2	+0.006	1.62 × 10^−4^	***	Higher

**Table 3:** Key gene–cell-type Spearman’s correlations from xCell deconvolution. All significant at *p*.adj < 0.05. The complete 56-correlation matrix across all genes and cell types is provided in Supplementary Materials. Statistical significance is indicated by asterisks: * (*p* ≤ 0.05); ** (*p* ≤ 0.01); *** (*p* ≤ 0.001).

Gene	Cell Type	Datasets (rho)	Sig.
*NLRP3*	Monocytes	GSE13904: +0.633	***
*NLRP3*	Neutrophils	GSE13904: +0.537	***
*GCLM*	Macrophages M1	GSE54514: +0.356/GSE13904: +0.366	*/***
*GCLM*	CD8+ T-cells	GSE54514: −0.490/GSE13904: −0.247/GSE28750: −0.455	**/*/*
*GCLC*	Monocytes	GSE54514: −0.530	***
*GCLC*	Neutrophils	GSE13904: −0.340	**
*SLC25A39*	Monocytes	GSE13904: −0.400	***
*SLC25A39*	Macrophages M1	GSE13904: −0.230	***
*SLC25A40*	Monocytes	GSE13904: +0.500	***
*NFE2L2*	Monocytes	GSE13904: +0.473/GSE54514: +0.415	***/*
*NFE2L2*	Neutrophils	GSE13904: +0.602	***
*AFG3L2*	Macrophages M1/M2	GSE28750: −0.740/−0.730	***
*GSDMD*	Macrophages M1	GSE13904: +0.379	***
*GSDMD*	Neutrophils	GSE13904: +0.253	*
*BNIP3L*	Monocytes	GSE54514: −0.451/GSE13904: −0.271	**/*
*BNIP3L*	Neutrophils	GSE13904: −0.310	**
*FUNDC1*	Macrophages M2	GSE13904: −0.501/GSE28750: −0.840	***/***
*FUNDC1*	B-cells	GSE13904: +0.494/GSE28750: +0.679	***/***
*FUNDC1*	CD8+ T-cells	GSE13904: +0.395/GSE28750: +0.844	***/***

**Table 4:** Theoretical taxonomy of DIC subtypes by predicted GSH-axis relevance. Classification generates testable predictions for cross-subtype validation (Experiment 5).

DIC Subtype	Primary Mechanism	Predicted GSH Axis Relevance
Sepsis-induced	Endothelial oxidative destruction, *NLRP3*/*GSDMD* pyroptosis, complement	HIGH, primary subject of this hypothesis
Cancer-associated	Mixed: endothelial oxidative stress and procoagulant protein release	HIGH to MODERATE (companion paper)
Obstetric (AFE)	Massive complement and endothelial activation	HIGH, oxidative-endothelial dominant
Major trauma	Endothelial injury, hypoperfusion-driven oxidative stress	MODERATE to HIGH
Genetic thrombophilia	Regulatory protein structural dysfunction	LOW, non-oxidative mechanism
Heparin-induced (HIT)	Immune-mediated platelet activation	LOW, immunological, non-oxidative
TTP/HUS	ADAMTS13 deficiency or complement dysregulation	LOW to MINIMAL

**Table 5:** Characteristics of the two adult and one pediatric whole-blood GEO datasets.

Dataset	Study	n Sep	n Ctrl	Platform	Notes	Samples
GSE54514	Parnell et al. [41]	35	18	Illumina HT-12	Septic shock; whole blood	53
GSE13904	Wong et al. [42]	99	18	Affymetrix U133 + 2	Pediatric; survivors and non-survivors (SIRS excluded)	117
GSE28750	Sutherland et al. [43]	10	20	Affymetrix U133 + 2	Adult ICU whole blood	30
Total	3 transcriptomic cohorts	144	56	2 platforms		200

## Data Availability

GEO datasets GSE54514, GSE13904, GSE28750, and GSE46955 are publicly available at https://www.ncbi.nlm.nih.gov/geo/ (accessed on 28 July 2026). The full analysis code, alongside the sample selection matrices to reproduce this study, is deposited on GitHub (https://github.com/felixbadiu9/The-enzymatic-mitochondrial-import-bottleneck-in-Sepsis, accessed on 28 July 2026) and archived in the Supplementary Materials.

## Supplementary Materials

The following supporting information can be downloaded at: https://www.mdpi.com/article/10.3390/ijms27198642/s1.
